# Indirubin, a small molecular deriving from connectivity map (CMAP) screening, ameliorates obesity-induced metabolic dysfunction by enhancing brown adipose thermogenesis and white adipose browning

**DOI:** 10.1186/s12986-020-00440-4

**Published:** 2020-03-16

**Authors:** Gang Wei, Honglin Sun, Jun-li Liu, Kai Dong, Junli Liu, Min Zhang

**Affiliations:** 1 Department of Endocrinology and Metabolism, Shanghai Diabetes Institute, Shanghai Jiao Tong University Affiliated Sixth People’s Hospital, Shanghai Jiao Tong University School of Medicine, Shanghai, 200032 People’s Republic of China; 2 Henan Key Laboratory of Neurorestoratology, Henan International Joint Laboratory of Neurorestoratology for Senile Dementia, The First Affiliated Hospital of Xinxiang Medical University, Weihui, 453100 Henan Province People’s Republic of China; 3 Department of Urology, Changzheng Hospital, Second Military Medical University, Shanghai, 200003 People’s Republic of China; 4 Shanghai Chest Hospital, Shanghai Jiaotong University, Shanghai, 200030 People’s Republic of China

**Keywords:** Connectivity MAP, Brown adipose tissue, Energy expenditure, Indirubin, Obesity

## Abstract

**Background:**

Obesity occurs when the body’s energy intake is constantly greater than its energy consumption and the pharmacological enhancing the activity of brown adipose tissue (BAT) and (or) browning of white adipose tissue (WAT) has been considered promising strategies to treat obesity**.**

**Methods:**

In this study, we took a multi-pronged approach to screen UCP1 activators, including in silico predictions, in vitro assays, as well as in vivo experiments.

**Results:**

Base on Connectivity MAP (CMAP) screening, we obtained multiple drugs that possess a remarkably correlating gene expression pattern to that of enhancing activity in BAT and (or) sWAT signature. Particularly, we focused on a previously unreported drug-indirubin, a compound obtained from the Indigo plant, which is now mainly used for the treatment of chronic myelogenous leukemia (CML). In the current study, our results shown that indirubin could enhance the BAT activity, as evidenced by up-regulated *Ucp1* expression and enhanced mitochondrial respiratory function in vitro cellular model. Furthermore, indirubin treatment restrained high-fat diet (HFD)-induced body weight gain, improved glucose homeostasis and ameliorated hepatic steatosis which were associated with the increase of energy expenditure in the mice model. Moreover, we revealed that indirubin treatment increased BAT activity by promoting thermogenesis and mitochondrial biogenesis in BAT and induced browning of subcutaneous inguinal white adipose tissue (sWAT) of mice under HFD. Besides, our results indicated that indirubin induced UCP1 expression in brown adipocytes, at least in part, via activation of PKA and p38MAPK signaling pathways.

**Conclusions:**

Our results clearly show that as an effective BAT (as well as beige cells) activator, indirubin may have a protective effect on the prevention and treatment of obesity and its complications.

## Background

The prevalence of obesity has been progressively rising worldwide over the past two decades, reaching pandemic levels [1]. According to the World Health Organization (WHO), in 2030, over one billion people in the world will be affected by obesity [2]. Obesity is one of important risk factors for metabolic diseases such as type2 diabetes mellitus, non-alcoholic steatohepatitis, cancers, etc., all of which contributes to a decline in both life quality and lifespan [3–5]. Obesity occurs when the body’s energy intake constantly exceeds its energy consumption. At present, the available drugs to treat obesity are mainly through limiting energy intake, including inhibiting intestinal lipid absorption (such as orlistat) or inhibiting appetite (such as phenylalanine) [6]. Though these medications are effective, the adverse side effects (such as steatorrhea or depression) due to long-term use limit drug adherence of patients. Therefore, there is an urgent need for safer and more effective pharmacological approaches to weight loss.

Obesity is defined as a state of excessive or abnormal fat accumulation of sufficient magnitude which may pose a threat to the health of people [4, 7]. However, mammals, in fact, possess two kinds of adipose tissue with distinct physiological functions: white adipose tissue (WAT) and brown adipose tissue (BAT). WAT stores excess energy in the form of triglycerides. In contrast, BAT increases energy expenditure by dissipating chemical energy as heat (thermogenesis), potentially counteracting obesity and related disorders [8]. Indeed, recent researches have shown that BAT transplantation could reduce the body weight gain and ameliorate glucose homeostasis in leptin deficient (ob/ob) obese mice and high-fat diet (HFD) -induced obese mice [9, 10]. Importantly, BAT transplantation in polycystic ovary syndrome (PCOS) rats also exhibited a significantly improvement in the key features of PCOS by increasing energy expenditure [11]. Recently, a number of studies have proved that adult humans possess functional BAT and its activity is negatively correlated with body mass index [12–14]. In humans, activation of BAT alleviates obesity, and decreases elevated plasma triglyceride concentrations [5].

An important feature of BAT is the expression of uncoupled protein 1 (UCP1), which is located in the inner membrane of the mitochondria [15–17]. In response to external stimuli (such as cold), UCP1 can increase the permeability of the inner mitochondrial membrane, during which UCP1 increases heat production instead of ATP synthesis [18–20]. As compared to other UCP proteins (e.g., UCP2 or UCP3), UCP1 is thought to be the only gene responsible for adaptation to non-shivering heat production [21, 22]. UCP1-null mice display intolerant to cold [23–25] and develop obesity housed at a thermoneutral temperature [26, 27]. By contrast, transgenic expression of UCP1 in adipose tissues reduces fat deposition and improves energy metabolism in rodents or pigs (in which a functional UCP1 gene is absent) [28–30].

Recently, a group of brown-like fat cells have been identified in white adipose tissue exposed to various stimulation (e.g., the cold), which are called “brite (brown in-white) or beige” adipocytes [31]. This process is called the “browning” of WAT. Similar to adipocytes in BAT, beige cells also express UCP1 protein (though at a lower level), resulting in increased mitochondrial respiration and energy expenditure. Correspondly, a pharmacological approach to increase UCP1 expression and activates of BAT thermogenesis and (or) recruits brown-like brite/beige cells in WAT may be a safer avenue to enhance whole-body energy expenditure, one complementary and alternative medicine for anti-obesity therapy [32, 33].

The Connectivity map (CMAP) is developed by the Broad Institute of MIT and Harvard University which is a web-based tool and allows users to screen of molecules for a physiological or disease gene signature [34–36]. Both drugs and diseases have so-called genetic signatures which consist of sets of genes known to be turned up or down, on or off in cells exposed to a particular drug or in patients with a particular disease. This screening is obtained by comparing microarray gene expression data of small compounds in CMAP database with the gene signature of the phenotype based on the user’s interest using a pattern-matching algorithm. By this way, a list of compounds exhibits in the results, which shown significantly correlating gene expression patterns as compare to that within the interest phenotype, thus facilitating potential treatments for disease in a specialized search engine. Since its publication, CMAP has been widely used and become one of the most effective tools for drug repositioning (also known as drug repurposing) and compounds screening to treat associated diseases. In recently studies published in *Nature Med* and *Cell*, Ozcan’s group of Harvard Medical School employed the CMAP database to identify candidate drugs to reduce ER stress in obesity. In this context, they found that withaferin A and celastrol as leptin sensitizers, can reduce the body weight and mitigate the metabolic abnormalities of diet-induced obesity mice, including hepatic steatosis [37, 38]. Besides, there were some other examples of CMAP used for identifying chemicals and (or) in combination with other therapies in treating T2D diabetes, muscular atrophy, inflammatory bowel disease and cancer [39–45]. Recently, as part of the NIH LINCS Consortium, more than a 1000-fold scale-up of the CMAP (termed L1000) was made [46, 47]. The expanded CMAP will play a greater role in exploring the relationships among drugs, genes, and diseases.

In our current study, we have created a gene expression signatures by utilizing the genes data obtained from CITGeneDB. CITGeneDB is a comprehensive database including genes of enhancing or suppressing Cold-Induced Thermogenesis (CIT) in human and mouse, which are validated by in vivo or ex vivo experiments in mice [48]. CITGeneDB has been integrated into Gene Ontology (GO) and MGI Mammalian Phenotype Ontology and can facilitate the research of CIT with systems biology perspectives. Based on CMAP database, we then used the signature to identify small compounds which could upregulate the UCP1 expression and enhanced activity in adipose (BAT and WAT), and thereby offer a treatment for obesity. In the current study, we paid particular attention to indirubin, a compound obtained from the Indigo plant a compound obtained from the Indigo plant, which is now mainly used for treating chronic myelogenous leukemia (CML). Our results indicate that as an effective BAT (as well as beige cell) activator, indirubin may have a protective effect on the prevention and treatment of obesity and related diseases, which involved in the up-regulation of UCP1 expression and enhancing the BAT activity and (or) inducing browning of sWAT, at least in part, via activation of PKA and p38MAPK signaling pathways.

## Materials and methods

### Chemicals and antibodies

The chemical compounds derived from CMAP screening were purchased from Chemical Biology Technology Platform of Chinese Academy of Sciences (shanghai, china) and applied in a dose of 10 μM (final concentration) for each drug within cell experiments. Indirubin used in animal treatment was purchased from MedChemExpress (Catalog No.: HY-N117; CAS No.: 479–41-4; Purity: >98%, Monmouth Junction, NJ). Indirubin was first dissolved in dimethyl sulfoxide (DMSO) and stored in the dark at − 20 °C. During our experiments, indirubin was diluted into corresponding concentration with the Corn oil (Sigma-Aldrich). All the other chemicals were purchased from Sigma Chemical Co. (St. Louis, MO, USA) unless otherwise specified. The primary antibodies included anti-UCP1 (Abcam, ab10983 and ab23841), anti-PGC1α (Santa Cruze Biotechnology, sc-13,067), anti-OXPHOS (Abcam, ab110413), anti- phospho-(Ser/Thr) PKA Substrate (Cell Signaling Technology, #9621), anti-phospho-CREB (Cell Signaling Technology, #4276S, San Antonio, TX, USA), anti-VDAC1 (Cell Signaling Technology, #4661), anti-β-Tubulin (Cell Signaling Technology, #2146).

### Cell culture, brown adipocytes differentiation, and function analysis

C3H10T1/2 cells were purchased from shanghai institute of biochemistry and cell biology (Shanghai, China) and routinely maintained in high glucose DMEM containing 10% fetal bovine serum (v/v), 100 U/ml penicillin, and 100 μg/ml streptomycin at 37 °C in a humidified atmosphere of 5% CO_2_ in air. For induction to brown adipocytes, the cells were incubated with medium that contained 20 nM insulin, 1 nM 3, 3, 5-triiodo-L-thyronine (T3), 1 μM dexamethasone, 0.5 mM isobutylmethylxanthine, 125 nM indomethacin, and 1 μM rosiglitazone for two days and were subsequently cultured in medium containing with only insulin and T3 for an additional 4 days. For the assessment of cellular respiration, O_2_ consumption of mature adipocytes were measured at day 6 using an XF24–3 Extracellular Flux Analyzer according to the manufacturer’s instructions (Agilent Technologies, Santa Clara, CA, USA). Cells treated with DMSO were used as control.

### Connectivity MAP analysis and cell viability assay

For the next-generation CMAP (https://clue.io) analyses, a gene expression signature (termed enhancing activity in BAT and (or) sWAT signature) was created by using the genes profile obtained from CITGeneDB [48]. The online tool of ‘Query’ was then used to directly explore the similarity between gene signatures of drugs and query samples with the query parameters of ‘Gene expression (L1000)’, ‘Touchstone’ and ‘Individual query’. For the prediction of novel drugs that enhance the activity of adipose in BAT and (or) browning of WAT, the genes of enhancing or suppressing cold-induced thermogenesis in human and mouse were used as the UP-regulated genes or DOWN-regulated genes, respectively. Drugs similarity was ranked according to the CMAP connectivity score (ranging from − 100 to 100) and displayed as a form of heat map, followed the ‘perturbation type’ were set as ‘compound’. The cell count kit-8 (CCK-8) assay was used for evaluating cell viability as described previously [49]. In brief, C3H10T1/2 cells were treated with indirubin or other compounds at the indicated concentrations either for 48 h in the undifferentiated state or for the entire differentiation process (until day 6). The absorbance was measured at a wavelength of 450 nm on a plate reader (Perkin Elmer, Waltham, MA, USA).

### Animals and treatments

Four-week-old C57BL/6 male mice were obtained from Shanghai SLAC Laboratory Animal Company and were housed four to five per cage under constant environmental conditions (12 h/12 h light-dark cycle, temperature 22 ± 2 °C, relative humidity at 50 ± 15%) in an office of Laboratory Animal Welfare-certified animal facility. After acclimatization for one week, the mice were randomly divided into different groups fed with either normal chow diet (NCD) or high fat diet (HFD, 60% of energy from fat, Research Diets Inc., cat.no. D12492) for six weeks. Then, mice were treated with indirubin (3.5 mg/kg) or vehicle (Corn oil, Sigma-Aldrich) as control by intraperitoneal injection (once every two days) for another 8 weeks while simultaneously fed NCD or HFD, respectively. Water and food were provided ad libitum unless otherwise specified. The body weight was assessed every Wednesday morning, and the food intake (mean daily food consumption) was measured at the tenth week by calculating the amount of food consumed at 24-h intervals for 6 days. After a 7-week treatment with either indirubin or vehicle, the total fat and lean masses of mice were tested with miniSpec NMR instrument (BCA-Body Composition Analyzer, Bruker Corporation, Billerica, MA, USA). At the end of the experiments, the blood plasma samples were collected and the weights of liver tussues and adipose tissues including BAT, subcutaneous inguinal white adipose tissue (sWAT) and epididymay white adipose tissue (eWAT) were measured. Tissues were preserved for gene expression, western blot analyses, histology, and immunohistochemistry experiments. All animal procedures in this study were performed in accordance with guideline from the Animal Care Committee of Shanghai Jiao Tong University.

### Metabolic analyses

Whole-body oxygen consumption was assesed with TSE lab master system (TSE Systems, Bad Homburg, Germany), as described previously [50]. Briefly, mice were maintained in respiration chambers (22 ± 2 °C, a 12-h light/dark cycle) with free access to food and water. Mice were adapted in the metabolic chambers for 24 h, and then VO_2_, VCO_2_, and physical activity were monitored during the next 24 h. Heat production and respiratory exchange ratio (RER) were then calculated [51].

### Glucose and insulin tolerance tests

For glucose tolerance test (GTT), mice had free access to drinking water and were fasted overnight (16 h, 5:00 PM to 9:00 AM). The fasting blood glucose and body weight of each mouse was recorded followed by an intraperitoneally (i.p.) injection of D-glucose (Sigma-Aldrich, 10% in saline, 1.5 g/kg body weight). Insulin tolerance tests were conducted in mice fasted 5 h (9:00 AM to 2:00 PM) by i.p. injection of insulin (0.75 IU/kg body weight, Novolin, USA). Blood glucose levels were detected with an Accu-Chek glucose monitor (Roche Diagnostics, Indianapolis, IN, USA) at 0, 15, 30, 60, 90, and 120 min.

### Biochemical analysis for serum samples and liver samples

Alanine aminotransferase (ALT), aspartate aminotransferase (AST), total triglyceride (TG), high-density lipoprotein (HDL-c), low-density lipoprotein (LDL-c), total cholesterol (TC) in serum were determined using commercial standard enzymatic assay kits (Nanjing Jiancheng Bioengineering Institute, Nanjing, Jiangsu, China). Liver TG and TC were measured by using commercially available assay kits (Applygen Technologies Inc., Beijing, China) and further normalized to protein concentration. Levels of insulin in the serum were measured using High sensitive mouse insulin immunoassay kit (AIS, cat.no. 32270) following the manufacturer’s instructions.

### Histology and immunohistochemistry

For histological analysis, tissues were fixed with 4% paraformaldehyde and embedded in paraffin. The sections were then prepared and stained with hematoxylin and eosin (Sigma) to observe the general morphological features. Adipocyte area was determined using a microscope (OLYMPUS BX51, Japan). At least 5 fields from random sections of each mouse sample were quantified, and the mean value was calculated. Immunohistochemistry staining was performed using rabbit anti-UCP1 antibody (at 1:500 dilution, Abcam, ab10983) as described previously [52].

### RNA isolation and real-time quantitative polymerase chain reaction (RT-qPCR)

Total RNA was extracted from tissues or cells with Trizol reagent (Invitrogen, Carlsbad, CA, USA). Reverse transcription of total RNA (500 ng) and RT-qPCR analysis were conducted with commercial kits (Vazyme Biotech, Nanjing, Jiangsu, China). RT-qPCR analysis was performed in duplicate for each sample and repeated three times utilizing a Roche LightCycler 480 system (Roche Diagnostics, Mannheim, Germany). Cyclophilin A was used as a control. Primer sequences used are listed in Supplymentary Table 1.

### mtDNA content quantification

For quantification of mtDNA, total DNA was isolated from BAT, sWAT and differentiated C3H10T1/2 cells with TIANamp Genomic DNA Kit (Tiangen Biotech Co., Ltd., Beijing, China). Quantitative PCR was performed in duplicate with mtDNA specific primer (COX II) and nuclear-specific PCR (β-globin). Primers used in qPCR analysis were designed from the published primer sequences by Yuan et al. [50]. The results were shown as abundance ratio of mtDNA target gene COXII to β-globin, which correspondingly represented mitochondrial DNA and genomic DNA, respectively.

### Western blot analysis

Protein from tissues or cells was extracted by RIPA buffer (50 mM Tris, 150 mM sodium chloride, 0.1% SDS, 1.0% Triton X-100, 0.5% sodium deoxycholate) containing protease and phosphatase inhibitor cocktail (Roche Diagnostics, Rotkreuz, Switzerland). Extracts were spun down, and the cell debris were removed before analysis by Western blot. Equivalent samples (40–60 μg of protein in each well) were separated by SDS-PAGE and transferred to PVDF membrane (Millipore, Burlington, MA, USA). Membranes were blocked with 5% (w/v) fat-free milk in TBST buffer for 1 h and incubated with different antibodies overnight at 4 °C, followed by incubation with secondary antibodies for 1 h at room temperature. Blots were conducted using enhanced chemiluminescence reagents (Thermo Fisher Scientific) and detected in a luminescent image analyzer according to the manufacturer’s protocols (LAS-4000, Fujifilm, Tokyo, Japan).

### Statistical analysis

Data were expressed as mean ± SD. The statistical significance of differences was determined using either the Student’s unpaired t test (2-tailed) or One-way ANOVA followed by Bonferroni’s multiple comparison post hoc tests. A value of *p* < 0.05 was considered statistically significant.

## Results

### Identification of indirubin as a potential UCP1 activator

**Fig. 1:**
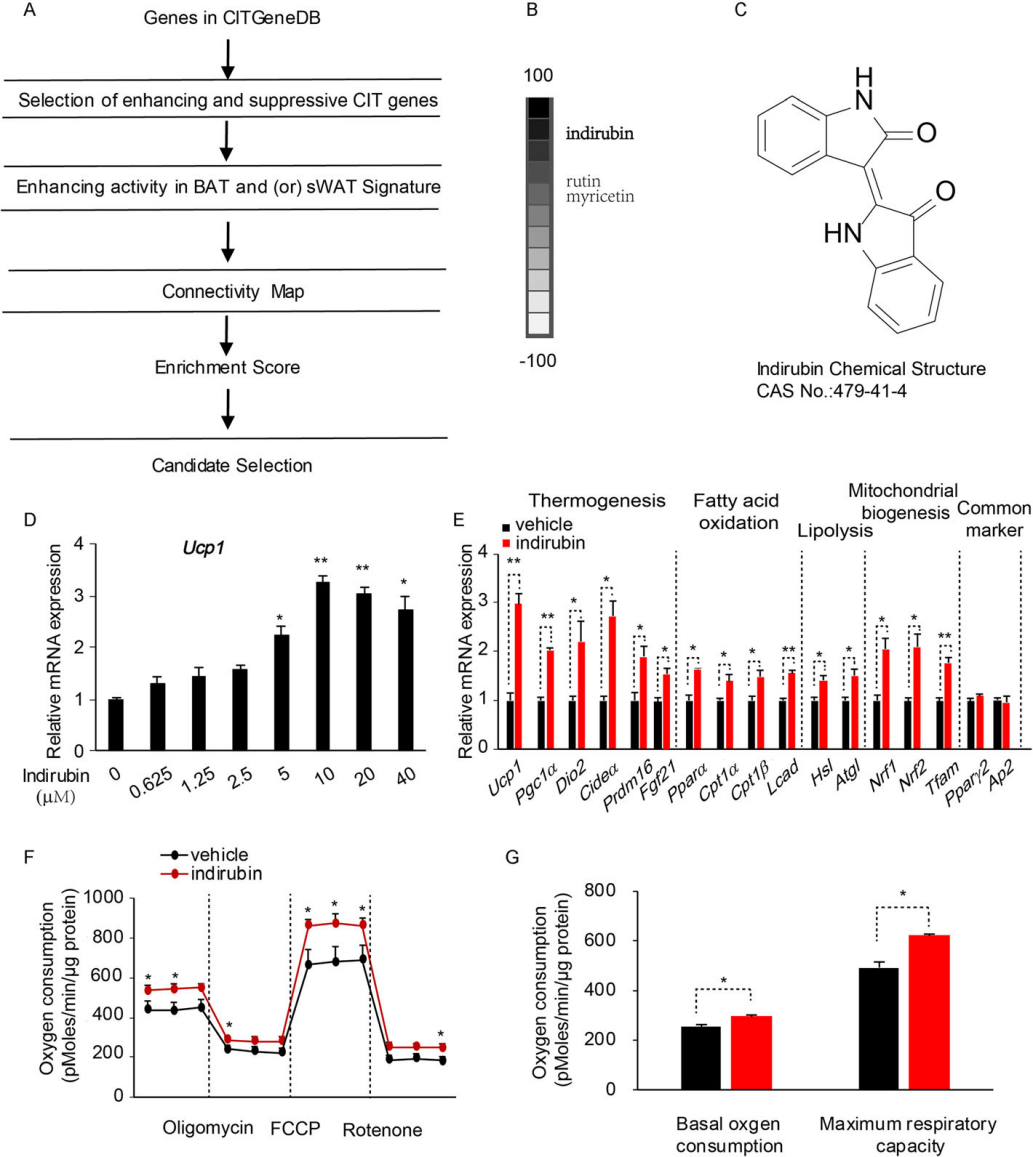
CMAP based screen identifies indirubin as a regulator of UCP1. **a** Flow chart depicting the process of CMAP screen using CITGeneDB to identify potential small-molecule for inducing UCP1. **b**, **c** The chemical structure and the CAS number of indirubin. **d** Dose-dependent effect of indirubin on *Ucp1* expression in differentiated C3H10T1/2 cells on day 6 of brown adipogenesis. **e** RT-qPCR analysis of genes related to thermogenesis, fatty acid oxidation, mitochondrial biogenesis and common adipogenic marks in differentiated C3H10T1/2 cells (6 days of differentiation) after indirubin (10 μM) treatment. **f** Oxygen consumption rates (OCR) was measured in differentiated C3H10T1/2 cells (6 days of differentiation) in the presence or absence of indirubin using a Seahorse XF24 analyzer. **g** Basal oxygen consumption and maximum respiratory capacity from seahorse assay were determined. Data in D-G are presented as mean ± SD of six independent experiments performed in duplicate. ^*^p < 0.05, ^**^p < 0.01 compared with vehicle

The CMAP links drugs with diseases or physiological phenotypes by using a pattern-matching algorithm and measuring similarities in gene expression [35, 46]. In this context, both drugs and diseases (and physiological phenotypes) have so-called genetic signatures which are sets of genes known to be turned up or down, on or off in a particular disease (or physiological phenotype) and in cells treatment by a particular drug. We hypothesized that perturbagen signatures of compounds in CMAP with corresponding signatures similar to that genetic signature of enhanced activity in BAT and (or) sWAT, will have good effects on improving obesity and obesity-related diseases. To test this hypothesis, we first created a gene expression signature (termed enhancing activity in BAT and (or) sWAT signature) from the gene profiles obtained from CITGeneDB (Fig. 1a, Supplymentary Table 2). Importantly, we chose the enhancing CIT genes as up-regulated genes and suppressive CIT genes as down-regulated genes. We then employed the query of CMAP for drugs which have a gene expression pattern positively correlating to the enhancing activity in BAT and (or) sWAT signature. During screening, multiple drugs in the CMAP were identified that had a significantly correlating gene expression pattern to that of enhancing activity in BAT and (or) sWAT signature, including rutin and myricetin (Fig. 1b). These results demonstrate the validity of the CMAP, because both rutin and myricetin can induce the UCP1expression in differentiated adipocytes, and enhance the thermogenesis of BAT and browning of sWAT in previous publications [50, 52].

We next set up a phenotype screen platform in vitro by examining the UCP1 expression and further oxygen consumption of differentiated C3H10T1/2 cells exposure to compounds derived from the CMAP. Herein, we focus on one drug of natural compounds derived from this model, which is named indirubin (Fig. 1c) and used as a Chinese medicine for the treatment of chronic myelogenous leukemia (CML) [53]. Prior to testing the effects of indirubin in adipocytes, we performed a CCK-8 assay in C3H10T1/2 cells to select optimal doses of indirubin. Indirubin has no significant cytotoxicity in concentrations up to 40 μM against the C3H10T1/2 cells for treatment of 48 h (Supplymentary Figure 1A). Moreover, indirubin did not show significant cytotoxicity when treated chronically (6 days) during differentiation (Supplymentary Figure 1B).

To assess the effect of indirubin on UCP1 expression, we differentiated C3H10T1/2 cells into adipocytes in the presence of several doses of indirubin for 6 days. At a dose of 5 μM and higher, indirubin markedly upregulated the expression of *Ucp1* mRNA (Fig. 1d). Consistently, indirubin (10 μM) treatment also increased the mRNA expression levels of thermogenic-related genes (*Pgc1α, Dio2, Cideα, Prdm16, and Fgf21*), FAO (fat acid oxidation) -related genes (*Pparα Cpt1α, Cpt1β, Lcad*), lipolysis-related genes (*Hsl, Atgl*) and mitochondrial biogenesis-related genes (*Nrf1/2, Tfam*) (Fig. 1e). However, indirubin treatment did not significantly affect the mRNA expression of common adipogenic marker genes *Pparg2* and *Ap2* (also known as *Fabp4*) (Fig. 1e). To determine the impact of indirubin on mitochondrial activities, oxygen consumption rate (OCR) was determined by the Seahorse system [54]. In agreement with gene expression data, indirubin increased the mitochondrial OCR, especially basal oxygen consumption as well as maximal respiration capacity (Fig. 1f-g). Therefore, these data show that indirubin may be a potential drug for combating obesity and obesity-related discords, in regard to its potential in inducing UCP1 expression and enhancing mitochondrial respiratory function in vitro.

### Indirubin protects against HFD-induced obesity

**Fig. 2:**
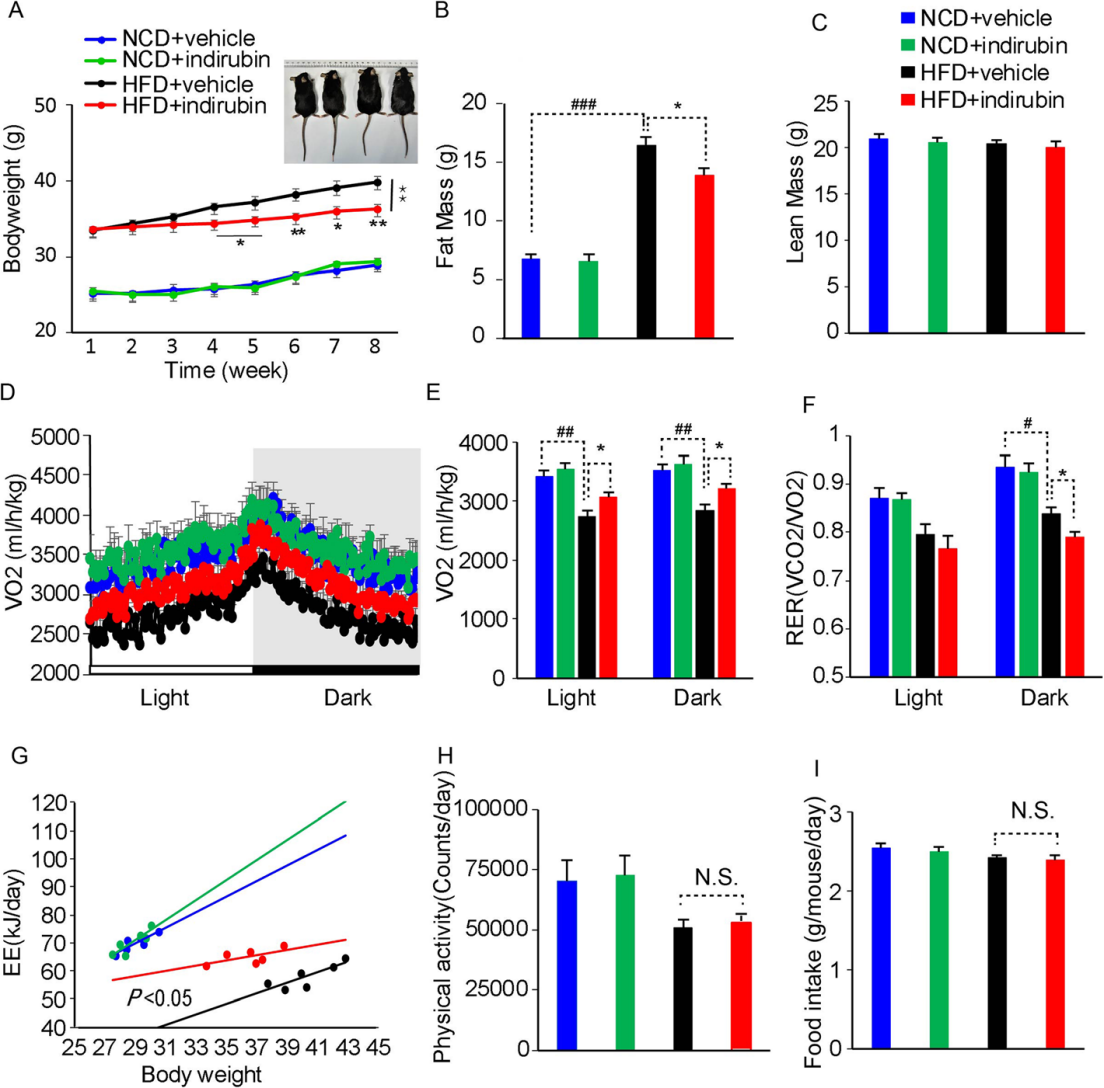
Indirubin treatment protects from HFD-induced obesity by increasing enhancing energy expenditure. **a** Body weights of mice were measured weekly during the last 8-week period (*n* = 6 in each group). Insets for A, representative images of mice at the end of 8-week experiment. (B-C) The total fat mass **b** and lean mass **c** were determined using miniSpec NMR instrument after 7-week treatment with either vehicle or indirubin. **d-h** Metabolic cage analyses of mice after 7-week treatment with either vehicle or indirubin (*n* = 6 in each group). The VO_2_ consumption during a 12-h light: 12-h dark cycle **d**, energy expenditure (EE), mean VO_2_ consumption levels **e**, the respiratory exchange ratio (RER; VCO_2_/VO_2_) **f**, and physical avtivity **h**, were measured simultaneously. **g** Energy expenditure values adjusted for body weight using ANCOVA. **i** Food intake (mean daily food consumption) was measured after 7-week treatment with either vehicle or indirubin by calculating the amount of food consumed at 24-h intervals for 6 days. Data are presented as mean ± SD (*n* = 6 in each group). ^#^*p* < 0.05, ^##^*p* < 0.01, ^###^*p* < 0.001 compared with NCD + vehicle group; ^*^p < 0.05, ^**^p < 0.01 compared with HFD + vehicle group

Next, we further assesed the effects of indirubin treatment in vivo. Mice were fed with normal chow diet (NCD) or high-fat diet (HFD) for six weeks, and then simultaneously treated with indirubin (or vehicle) at a dose of 3.5 mg/kg through intraperitoneal injection (once every two days) for another 8 weeks. When mice fed under NCD conditions, indirubin treatment had no obvious effects on body weight gain as well as body composition (Fig. 2a-c). However, indirubin treatment significantly inhibited the body weight gain in mice fed on a HFD (Fig. 2a), which was generally owing to the decreased fat mass (Fig. 2b-c). In addition, indirubin treatment did not remarkably affect the food intake in both NCD and HFD groups (Fig. 2i).

To examine whether indirubin-mediated decline in adiposity and body weight gain is associated with increased whole-body energy expenditure, mice were then housed in metabolic cages for monitoring O_2_ consumption after 6 weeks’ treatment of indirubin. On the normal chow diet, indirubin-treated mice exhibited comparable O_2_ consumption (Fig. 2d-e). When placed on HFD, indirubin-treated mice displayed significantly increased O_2_ consumption without much difference in physical activity (Fig. 2d-e, h), which suggested that the reduced body weights were not due to alteration in physical activity. Moreover, indirubin-treated mice displayed a higher reliance on fat oxidation, which was reflected by the relative lower-RER values (Fig. 2f). Because body weights were significantly different between vehicle- and indirubin-treated mice under HFD conditions, energy expenditure (EE) was statistically adjusted for body weight using analysis of covariance (ANCOVA). In line with increased O_2_ consumption, indirubin-treated mice exhibited greater EE than that of HFD group (Fig. 2g). These data suggest that indirubin treatment is resistant to HFD-induced obesity by increasing whole-body energy consumption.

### Indirubin improves whole-body glucose homeostasis, reduces lipid accumulation in adipose, and ameliorates fatty liver

**Fig. 3:**
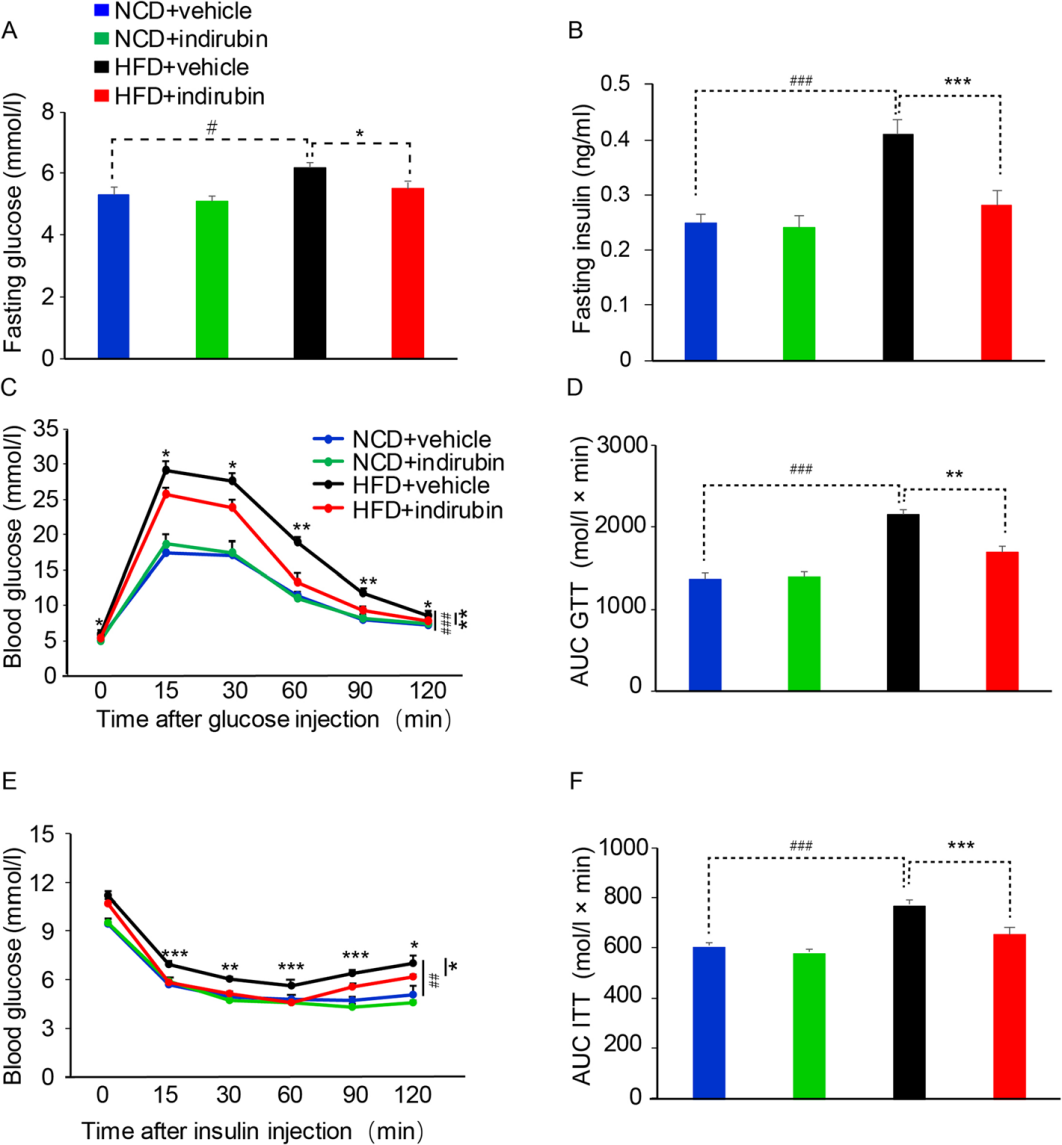
Indirubin treatment improves glucose tolerance and insulin sensitivity in HFD-induced obese mice. **a** Blood concentrations of glucose in mice fasted 16 h after 6-week treatment with either vehicle or indirubin. **b** Blood concentrations of insulin in mice fasted overnight after 8-week treatment with either vehicle or indirubin. **c** Glucose tolerance tests (GTT) performed in mice (*i.p*. injection of *glucose,* 1.5 g /kg) fasted 16 h after 6-week treatment with indirubin or vehicle. (*n* = 6 for each treatment). **d** Area under curve (AUC) for glucose based on data in **c**. **e** Insulin tolerance tests (ITT) performed in mice (*i.p*. injection of *insulin,* 0.75 U /kg) fasted 4 h after 7-week treatment with indirubin or vehicle. **f** Area under curve (AUC) for glucose based on data in **e**. Data are presented as mean ± SD (*n* = 6 in each group). ^#^*p* < 0.05, ^##^*p* < 0.01, ^###^*p* < 0.001 compared with NCD + vehicle group; ^*^p < 0.05, ^**^p < 0.01, ^***^p < 0.01 compared with HFD + vehicle group

As reduced adiposity is usually related to better glucose homeostasis, we next investigated if reduced fat mass in indirubin-treated mice under HFD would result in improved glucose disposal ability and insulin sensitivity. As our expected, fasting blood glucose levels and insulin levels were obviously lower in indirubin-treated mice under HFD (Fig. 3a-b), whereas corresponding fasting glucose levels and insulin levels were not significantly different between NCD groups (Fig. 3a-b). Consistent with these results, indirubin treated mice under HFD also displayed improved glucose handling as determined by glucose and insulin tolerance tests (at weeks 10 and 12, respectively) (Fig. 3c- f).

**Fig. 4:**
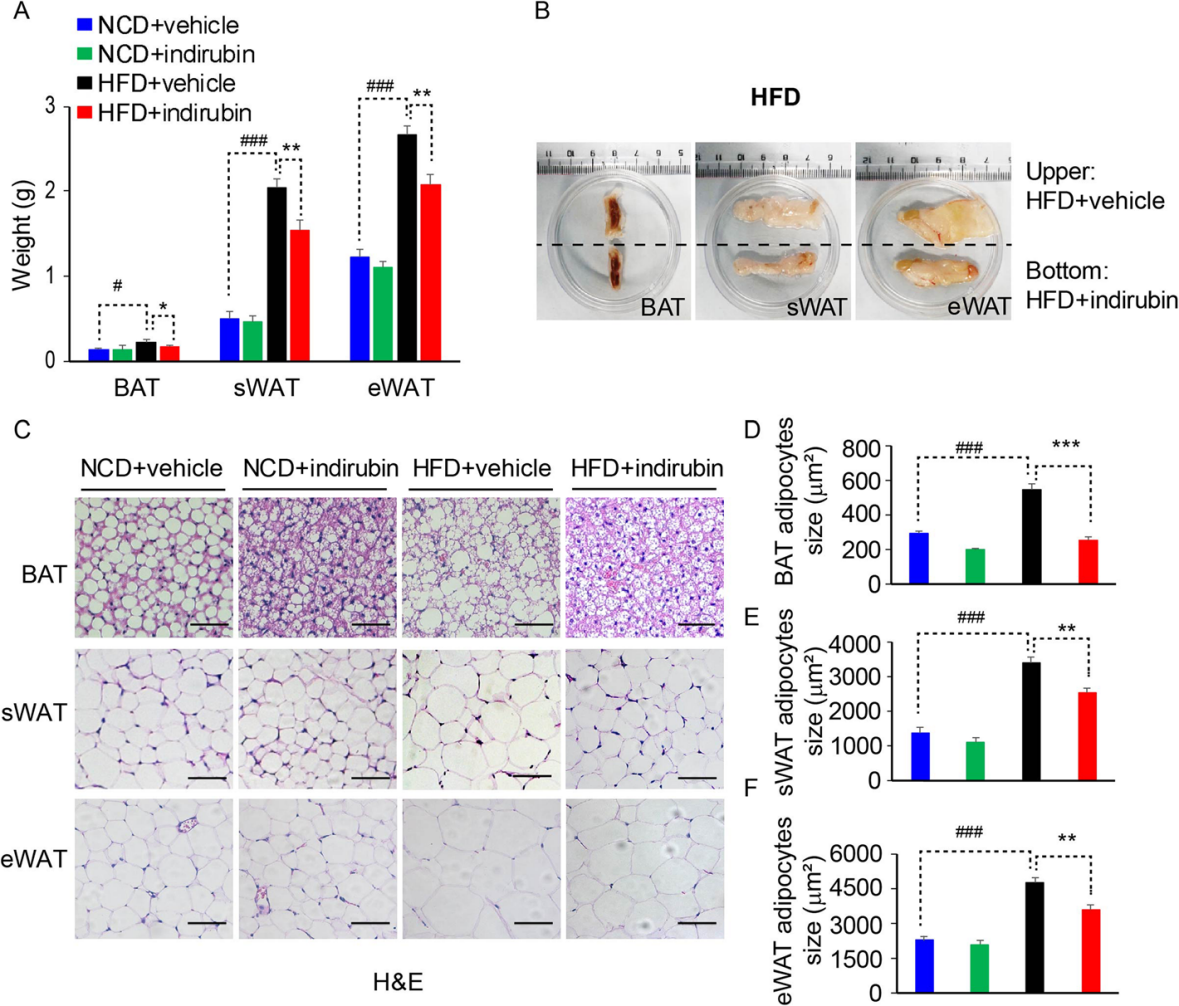
Indirubin treatment decreases adipose tissue mass and adipocyte cell size in HFD-induced obese mice. **a** Mean tissue weights of BAT, iWAT, and eWAT of mice fed NCD or HFD after 8-week treatment with either vehicle or indirubin. **b** Representive photographs of BAT, iWAT, and eWAT in HFD-induced obese mice after 8-week treatment with either vehicle or indirubin. **c** Representative hematoxylin and eosin (H&E) staining images of BAT, iWAT, and eWAT of mice fed NCD or HFD after 8-week treatment with either vehicle or indirubin. Scale bar = 50 μm. **d** Mean adipocyte size of adipose tissues quantified from H&E-stained section in **c**, six fields per mouse, using Metaxpress software. Data in **a**, **d**, **e** and **f** are presented as mean ± SD (*n* = 6 in each group). ^#^*p* < 0.05, ^###^*p* < 0.001 compared with NCD + vehicle group; ^*^p < 0.05, ^**^p < 0.01, compared with HFD + vehicle group

**Fig. 5:**
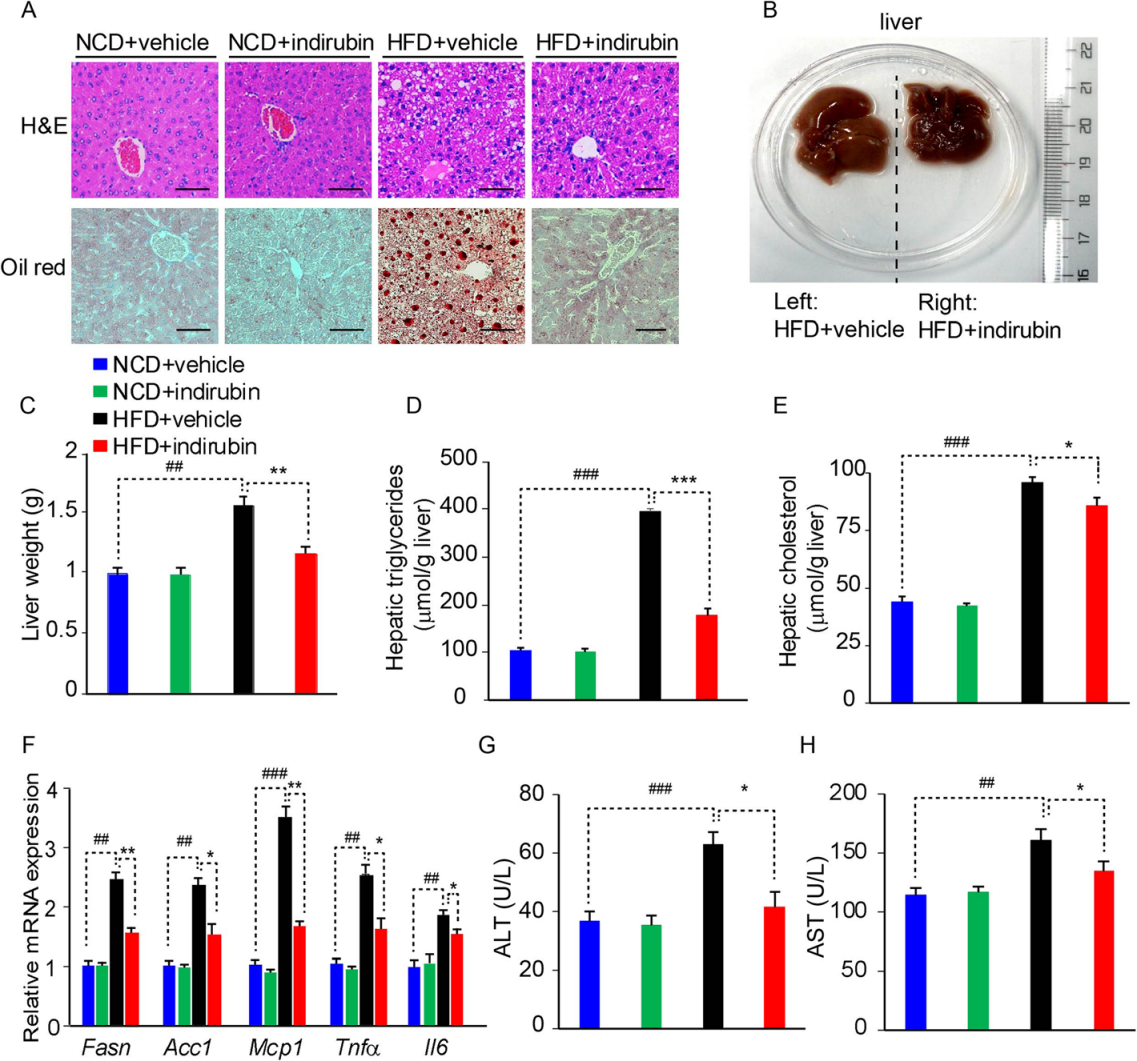
Indirubin treatment ameliorated hepatic steatosis in HFD-induced obese mice. **a** Representative H&E staining (upper) and Oil-red staining (lower) images of liver tissue of mice fed NCD or HFD after 8-week treatment with either vehicle or indirubin. Scale bar = 50 μm. **b** Representive photographs of liver tissue in HFD-induced obese mice after 8-week treatment with either vehicle or indirubin. **c** Mean tissue weights of liver were measured in mice fed NCD or HFD after 8-week treatment with either vehicle or indirubin. **d-e** Liver TG **d** and TC **e** content, serum ALT **g** and AST **h** were measured in mice fed NCD or HFD after 8-week treatment with either vehicle or indirubin. **f** RT- qPCR analysis of genes related to adipogenesis and inflammation in livers of mice fed NCD or HFD after 8-week treatment with either vehicle or indirubin. **g-h** Serum ALT **g** and AST **h** were measured in mice fed NCD or HFD after 8-week treatment with either vehicle or indirubin. Data in **c-h** are presented as mean ± SD (*n* = 6 in each group). ^##^*p* < 0.01, ^###^*p* < 0.001 compared with NCD + vehicle group; ^*^p < 0.05, ^**^p < 0.01, ^***^p < 0.01 compared with HFD + vehicle group

Mice were then dissected for further investigation. In line with the lean phenotype, total organ weights of BAT (− 26.09%), sWAT (inguinal WAT, − 24.80%) and eWAT (epididymal WAT, − 22.20%) were decreased in indirubin-treated mice of HFD groups but not in mice of NCD groups (Fig. 4a-b). Moreover, indirubin-treated mice under HFD exhibited remarkably decreased lipid content and adipocyte size in BAT, sWAT and eWAT as evidenced by histological analysis (Fig. 4c-f), which were correlated with body weight loss (Fig. 2a). In addition, the symptom of fatty liver induced during HFD was also alleviated by indirubin treatment, as assessed by morphological examination and histological analysis (Fig. 5a-b). After 14 weeks of HFD, indirubin-treated mice exhibited a significantly reduction in liver weights (Fig. 5c). Consistent with this result, indirubin treatment significantly ameliorated neutral lipid accumulation in livers of HFD-induced mice, as indicated both by means of H&E staining and Oil Red O analysis (Fig. 5a). In addition, indirubin-treated mice also displayed reduced levels of liver triglyceride (− 54.75%) and liver cholesterol content (− 10.66%) in (Fig. 5d-e) in livers of HFD-induced mice.

As a higher degree of obesity often leads to lipid metabolism abnormalities, we next evaluated the effects of indirubin on HFD-fed mice by measuring serum lipid metabolism-related biochemical parameters. As shown in Table 1, indirubin treatment prevented the HFD-induced elevation in the serum levels of TG, TC and LDL-c, whereas indirubin-treated mice exhibited considerably increase in the serum levels of HDL-c under HFD conditions. In line with these results, as shown in Fig. 5f, RT-qPCR assays also confirmed that indirubin treatment could reduce the mRNA expression levels of lipogenesis-associated genes (*Fans, Acc1*). Meanwhile, we found less expression of *Mcp1*, *TNF-α* and *IL-6* cytokines in liver tissues of mice after indirubin treatment under HFD (Fig. 5f), suggest an improved chronic inflammation state. Of note, we also analyzed serum levels of AST and ALT to determine whether indirubin treatment causes liver injury. As shown in Fig. 5g-h, compared with NCD groups, liver damage was verified by obviously elevated levels of serum AST and ALT in HFD-fed mice, whereas indirubin treatment protected against the increase in AST and ALT levels (Fig. 5g-h). These results indicated that indirubin treatment, at least in the concentration applied in this study, had no side effects on liver function. Taken together, these data show that indirubin treatment can improve glucose metabolism, reduce lipid accumulation in adipose and adipocyte size, as well as decrease hepatic fat deposition in HFD-fed mice.

**Table 1:** Plasma profiles

Parameters	NCD+ vehicle	NCD + indirubin	HFD+ vehicle	HFD + indirubin
TG (mM)	0.89 ± 0.04	0.81 ± 0.04	1.29 ± 0.02 ^#^	0.97 ± 0.07 ^*^
TC (mM)	1.87 + 0.05	1.78 ± 0.04	2.64 ± 0.08^##^	1.99 ± 0.17^**^
LDL-c (mM)	0.83 + 0.07	0.81 ± 0.07	1.37 ± 0.06^##^	0.88 ± 0.09^*^
HDL-c (mM)	1.53 + 0.04	1.55 ± 0.03	1.94 ± 0.14 ^#^	2.31 ± 0.16^*^

Plasma profiles of mice with or without indirubin treatment. Data are presented as mean ± SD (*n* = 6 in each group). #*p* < 0.05 compared with NCD + vehicle group;*p < 0.05, **p < 0.01 compared with HFD + vehicle group

### Indirubin increases BAT activity

**Fig. 6:**
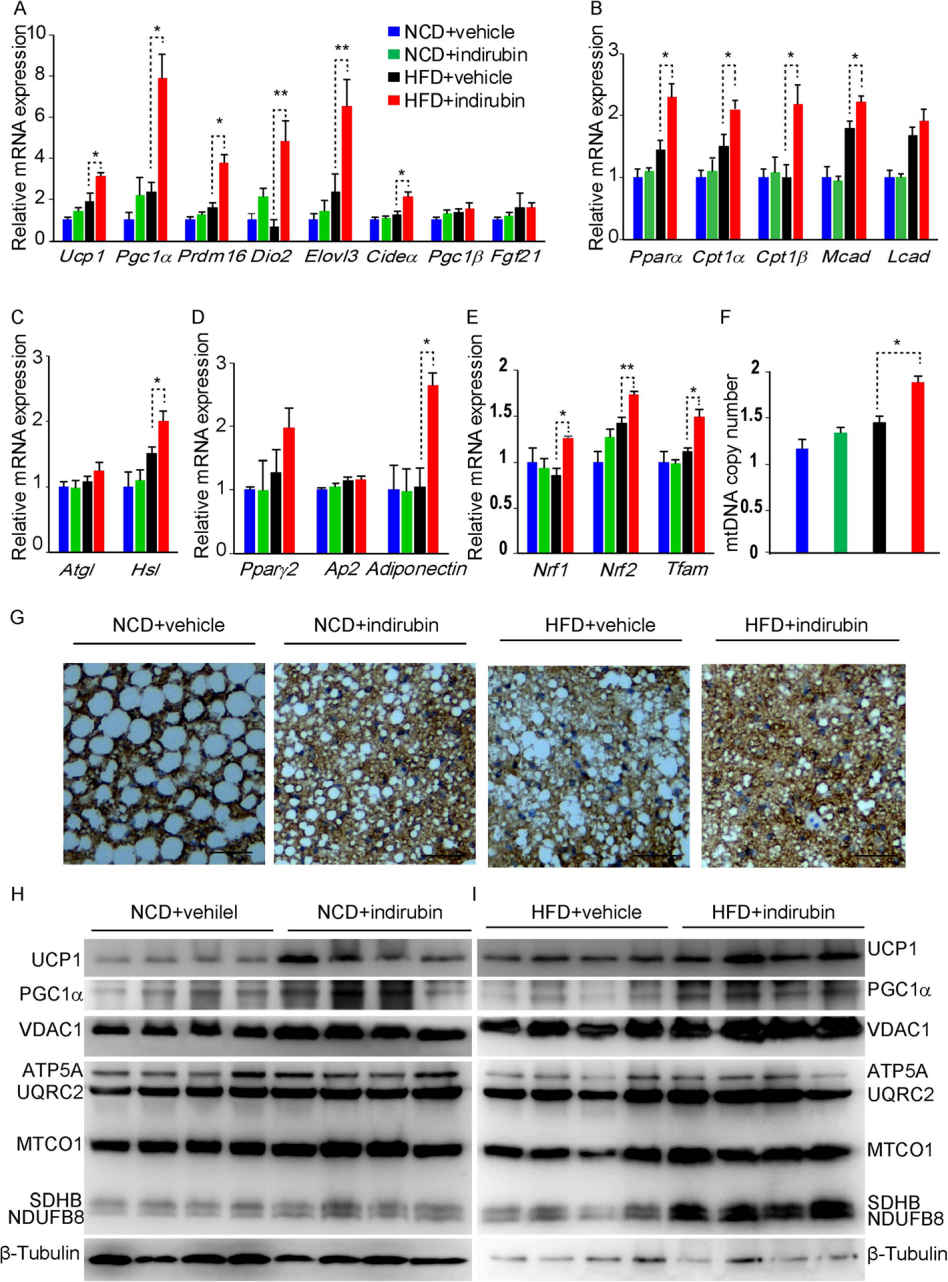
Indirubin increases BAT activity by promoting thermogenesis and mitochondrial biogenesis in HFD-induced obese mice. **a-e** RT-qPCR analysis of genes related to thermogenesis **a**, fatty acid oxidation **b**, lipolysis **c**, common adipogenic marks **d** and mitochondrial biogenesis in BAT of mice fed NCD or HFD after 8-week treatment with either vehicle or indirubin. **f** Measurement of mtDMA copy number in BAT of mice fed NCD or HFD after 8-week treatment with either vehicle or indirubin. **g** Immunohistochemistry (IHC) staining with a UCP1-specific antibody in BAT of mice fed NCD or HFD after 8-week treatment with either vehicle or indirubin. Scale bar = 50 μm. **h-i** Western blot analysis of proteins levels of UCP1, PGC1α, VDAC1, and OXPHOS in BAT of mice fed NCD **h** or HFD **i** after 8-week treatment with either vehicle or indirubin. β-tubulin serves as a loading control. Data in A-F are presented as mean ± SD (*n* = 6 in each group). ^*^p < 0.05, ^**^p < 0.01 compared with HFD + vehicle group

In order to investigate the close relationship of enhanced energy consumption to BAT activation, we examined the molecular biological features of BAT in mice after indirubin treatment. Consistent with enhanced whole-body energy consumption, the BAT marker UCP1 was upregulated significantly in indirubin-treated mice under HFD by immunohistochemical analysis (Fig. 6g). Furthermore, RT-qPCR analysis of BAT showed that indirubin treatment also led to upregulation of expression levels of BAT-specific genes related to heat generation, including *Ucp1, Pgc1α, Prdm16, Dio2, Elovl3, Cideα* (Fig. 6a). Meanwhile, the mRNA expression levels of genes controlling fatty acid oxidation (*Pparα, Cpt1α, Cpt1β, Mcad*) were significantly increased by indirubin treatment in BAT (Fig. 6b). In addition, the mRNA expression level of hormone-sensitive lipase (*Hsl*), which is closely related to triglyceride hydrolysis showed a slight but significant increase in indirubin-treated mice under HFD (Fig. 6c). However, as shown in Fig. 6d, common adipocytes makers (*Pparγ2, Ap2*) were not significantly affected after indirubin treatment, though the expression level of the insulin sensitizing adipokine adiponectin (also known as AdipoQ) was significantly higher relative to control HFD-fed mice. In addition, the BAT-enriched genes mentioned above showed no change or a slight increase in expression levels between NCD groups (Fig. 6a-d). These cumulative evidences indicated that indirubin had potential to increase the thermogenic capacity in BAT of HFD-fed mice.

Since the higher activity of BAT is often positive correlating to the number and activity of mitochondria. Therefore, we next measured the mRNA expression levels of mitochondriogenesis-related factors. As expected, *Nrf1/2* and *Tfam* expression levels were also upregulated (Fig. 6e). Consist with this, the number of mitochondria was increased in BAT of indirubin-treated mice under HFD as quantified by mitochondrial DNA (mtDNA) copy number (Fig. 6f). Furthermore, the protein abundance of voltage-dependent anion channel 1 (VDAC1), which is a major isoform highly and predominantly expressed on the mitochondrial outer membrane, was obviously upregulated after indirubin treatment in BAT of mice under HFD (Fig. 6i). In addition, the abundance of proteins (UCP1, PGC1α, and OXPHOS) related to thermogenesis and β-oxidative phosphorylation were markedly unregulated in BAT of indirubin-treated mice under HFD (Fig. 6i). However, BAT-enriched genes and (or) proteins mentioned above and mtDNA copy number showed no change or a slight increase in expression levels between NCD groups (Fig. 6a-h).

Taken together, these results indicate that indirubin can promote thermogenesis and mitochondrial biogenesis in BAT, thereby enhancing endogenous BAT activity and burning of fat.

### Indirubin induces browning of sWAT

**Fig. 7:**
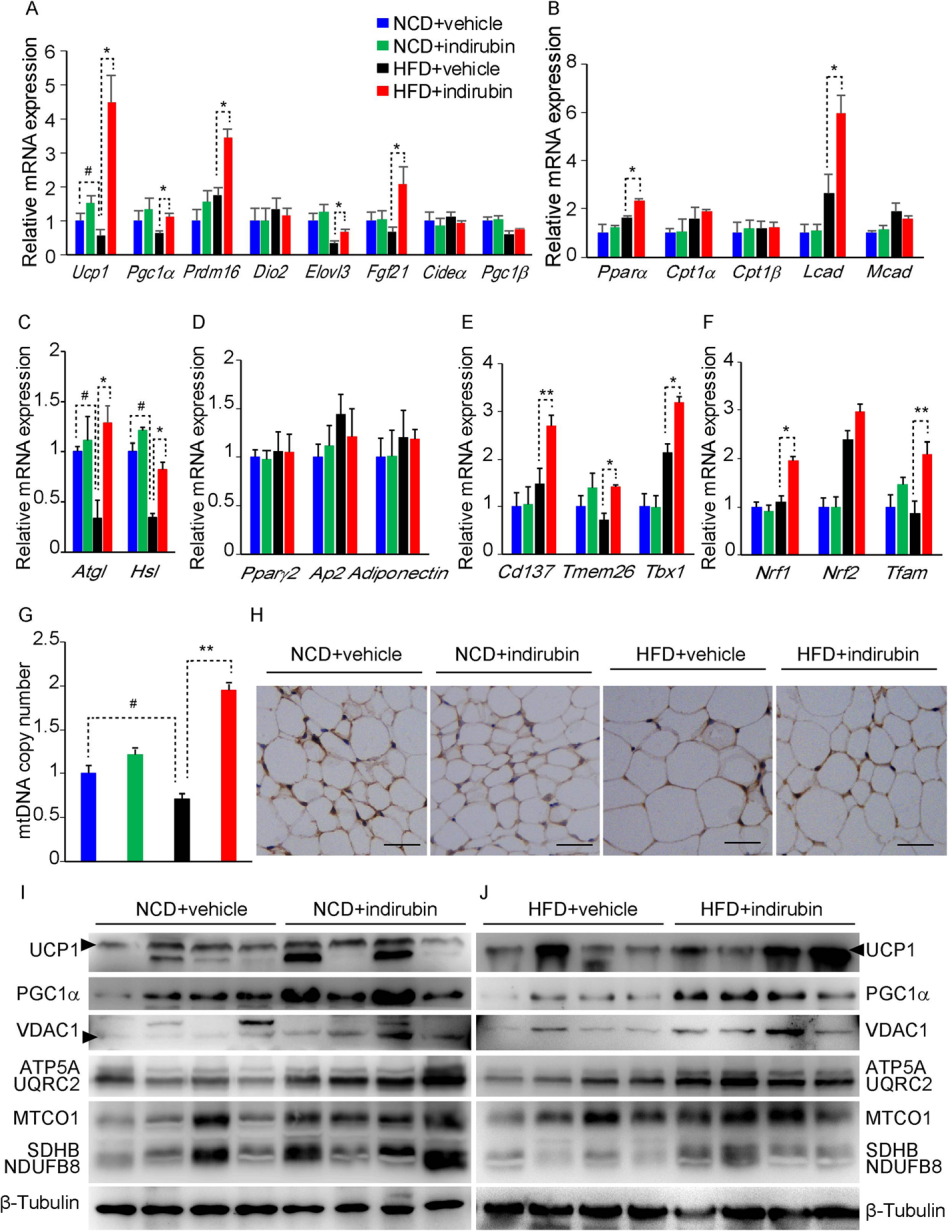
Indirubin promotes browning of sWAT by increasing BAT-specific markers and mitochondrial biogenesis in HFD-induced obese mice. **a-f** RT-qPCR analysis of genes related to thermogenesis **a**, fatty acid oxidation **b**, lipolysis **c**, common adipogenic marks **d**, beige cell markers **e** and mitochondrial biogenesis **f** in sWAT of mice fed NCD or HFD after 8-week treatment with either vehicle or indirubin. **g** Measurement of mtDMA copy number in sWAT of mice fed NCD or HFD after 8-week treatment with either vehicle or indirubin. **h** Immunohistochemistry (IHC) staining with a UCP1-specific antibody in BAT of mice fed NCD or HFD after 8-week treatment with either vehicle or indirubin. Scale bar = 50 μm. **i-j** Western blot analysis of proteins levels of UCP1, PGC1α, VDAC1, and OXPHOS in BAT of mice fed NCD (H) or HFD **i** after 8-week treatment with either vehicle or indirubin. β-tubulin serves as a loading control. Data in A-F are presented as mean ± SD (*n* = 6 in each group). ^#^*p* < 0.05 compared with NCD + vehicle group;^*^p < 0.05, ^**^p < 0.01 compared with HFD + vehicle group

Recent researches have proved that browning of sWAT can also increase energy metabolism and exhibit beneficial effects on anti-obesity [55–58]. To further assesed the reason of sWAT mass loss in indirubin-treated mice under HFD conditions, molecular biological characteristics of sWAT were researched. Interestingly, immunohistochemistry results indicated that UCP1 was strikingly stimulated in sWAT of mice under HFD in response to indirubin treatment. Besides, we next examined the BAT- enriched genes in sWAT (Fig. 7g). Our results showed that the mRNA expression levels of genes related to thermogenesis (*Ucp1, Pgc1α, Prdm16, Elovl3, Fgf21*) and fatty acid oxidation (*Pparα, Lcad*) were significantly upregulated in sWAT of mice treated with indirubin under HFD. Lipid droplets lipolysis in sWAT is essential for regulating BAT fuel sources and WAT thermogenesis during metabolic adaption [59, 60]. In our study, as shown in Fig. 7c, the mRNA expression levels of genes involved in lipolysis such as adipose triacylglycerol lipase (*Atgl*) and *Hsl* were obviously reduced in sWAT of HFD-fed control mice relative to NCD groups, whereas the mRNA expression levels of these two genes were considerably upregulated in sWAT of indirubin-treated mice under HFD, leading to the activation of lipolysis and fueling thermogenesis during BAT activation and sWAT browning. More importantly, beige cell marker genes (*CD137, Tmem26, Tbx1*) [61] were also markedly increased in sWAT of indirubin-treated mice as compared to HFD-fed control mice (Fig. 7e). However, there was no significant change in common adipose markers (*Pparγ2, AP2, Adiponectin*) at mRNA expression level under both diets (Fig. 7d). As beige cells has the characteristics similar to BAT, we also investigated the mitochondrial biogenesis in sWAT. the mRNA expression levels of *Tfam* and *Nrf1/2* in sWAT were notably upregulated after indirubin treatment under HFD (Fig. 7f). In parallel, indirubin treatment increased the number of mitochondria in sWAT, as was further manifest by increased mtDNA copy number (Fig. 7g) and mitochondrial outer membrane protein VDAC1 (Fig. 7j). Consistently, western blot analysis indicated that the abundance of proteins (UCP1, PGC1α, and OXPHOS) related to thermogenesis and β-oxidative phosphorylation were significantly unregulated in sWAT of indirubin-treated mice under HFD (Fig. 7j). These results indicate that increased browning of sWAT in response to indirubin can act synergistically with BAT activation on anti-obesity.

### Indirubin induces UCP1 expression and enhance BAT activity relying on PKA and p38MAPK signaling pathways

**Fig. 8:**
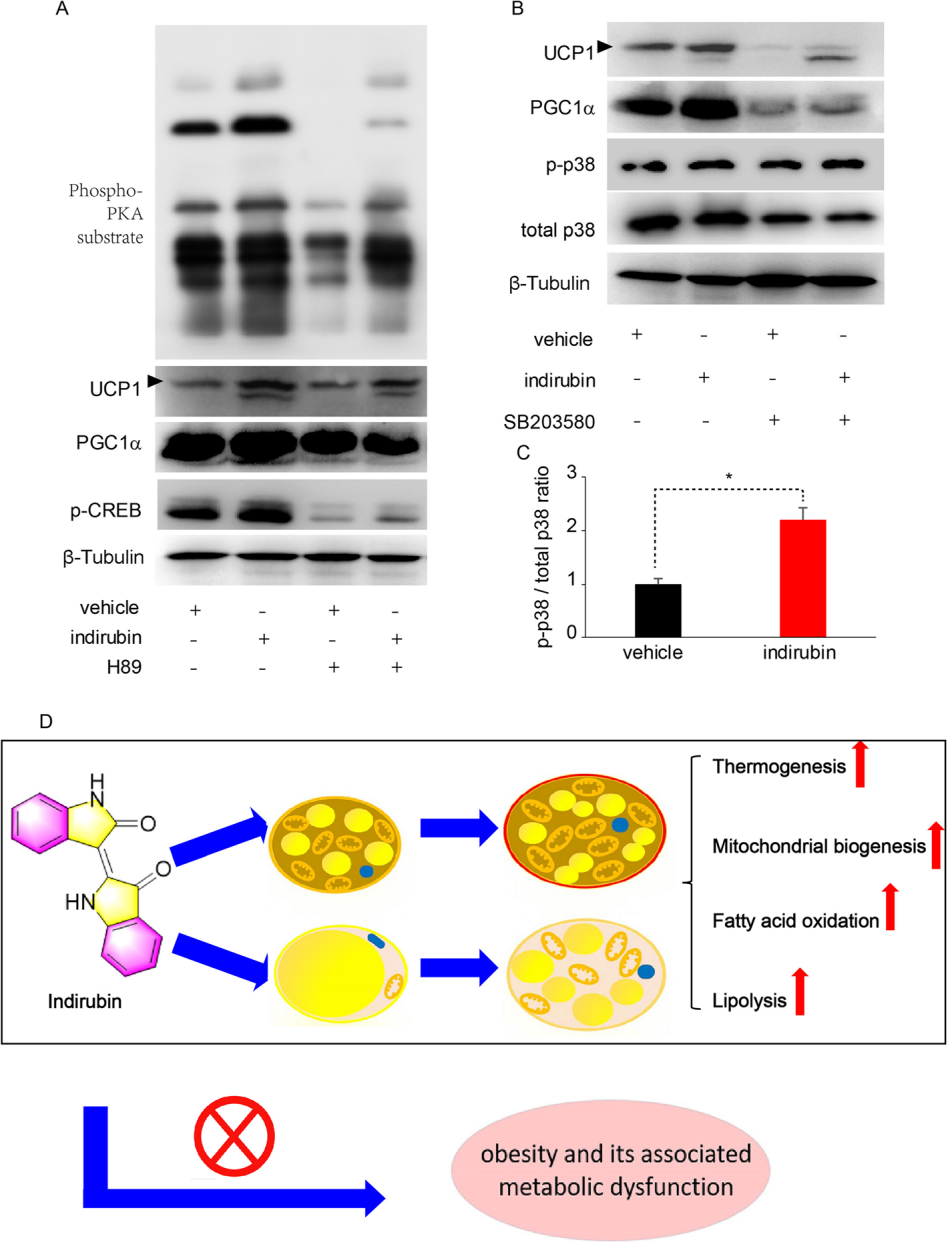
Indirubin enhances BAT activity and induces browning of WAT relying on PKA and p38MAPK signaling pathways. **a** Western blot analysis performed with the indicated antibodies (Phospho-PKA substrate, UCP1, PGC1α, p-CREB) in differentiated C3H10T1/2 cells on day 6 of brown adipogenesis in present or absent of indirubin and (or) PKA inhibitor. β-tubulin serves as a loading control. **b** Western blot analysis performed with the indicated antibodies (UCP1, PGC1α) in differentiated C3H10T1/2 cells on day 6 of brown adipogenesis in present or absent of indirubin and (or) p38 MAPK inhibitor. β-tubulin serves as a loading control. **c** Semi-quantitative analysis of p-p38/p38 ratio from (B). **d** Proposed model for the role of indirubin in activating BAT and browning of WAT, thus having benefit effects on ameliorating obesity and its associated metabolic dysfunction

To identify the possible mechanism underlying the effects caused by indirubin treatment, we determined expression levels of key signaling molecules involved in the regulation of brown adipose function. We investigated cAMP-dependent protein kinase A (PKA) and p38 MAPK signal pathways, both of which play important roles in the mobilization of fat to be hydrolyzed, oxidized, and uncoupled. Firstly, we used anti-phosphorylated PKA substrate antibody to evaluate PKA activity after indirubin treatment in differentiated adipocytes. As shown in Fig. 8a, indirubin treatment increased PKA activity as observed by increased phosphorylation of PKA substrate. CREB and p38 MAPK are important downstream targets of PKA, both of which have been proved as key elements in inducing UCP1 expression [19, 62]. In our current study, the increased protein abundance of phosphorylated forms of CREB and p38 MAPK were also observed by western blot analysis after treatment with indirubin compared with vehicle-treated cells (Fig. 8a-c). More importantly, we separately treated cells with PKA inhibitor (H89) and p38 MAPK inhibitor (SB203580) in the presence or obscene of indirubin at 6 days of differentiation. As shown in Fig. 8a, inhibition of PKA with H89 abolished indirubin-induced expression of BAT-enriched genes, including UCP1, PGC1α. Similarly, elevated expression of BAT-enriched genes (UCP1 and PGC1α) were eliminated after p38 MAPK inhibitor treatment (Fig. 8b). These results indicate that indirubin treatment can promote thermogenesis via activated PKA and p38 MAPK pathways. Collectively, these results suggest that indirubin may be a useful as herbal medicine for inducing UCP1 expression and enhance BAT activity (as well as browning of sWAT) via activation of PKA and p38MAPK signaling pathways, thereby potentially in preventing and treating obesity and obesity-associated metabolic diseases.

## Discussion

Obesity occurs when the body’s energy intake is constantly greater than its energy consumption, which has become a growing public health concern in modern society [4]. Since the recent discovery of functional BAT or BAT-like tissue in adult humans, the development and function of brown or beige adipocyte has been widely and deeply researched for its potential as a target of anti-obesity therapeutics [63]. Although many screening approaches (in vitro and in vivo) have been adopted for the discovery of chemicals to activate UCP1 expression [64–67], to identify safe and effective drugs for upregulating the activity of brown and (or) beige adipocytes is still a challenge, especially given the high-cost and time-consuming traditional methods for screening UCP1 activators. In order to address this challenge, we took a multi-pronged approach to screen UCP1 activators (through drug repurposing), including in silico predictions, in vitro assays, as well as in vivo experiments. In the in silico predictions, the CMAP database was employed to select appropriate drug candidates for further in vitro and in vivo validation. Our results indicate that indirubin is a promising natural product for protecting obesity and associated diseases through enhancing BAT activity and inducing browning of sWAT, at least in part, by activating PKA and p38 MAPK signal pathways.

In this work, we firstly used a gene profile obtained from CITGeneDB to construct the gene expression signature as enhancing activity in BAT and (or) sWAT signature. Because the genes of enhancing or suppressing CIT in CITGeneDB were all validated via perturbation experiments in mice, it will be more effective to highlight the role of core genes in regulation of the activity of brown or beige adipocytes. For example, the complete list of cold-modulated genes in BAT after microarray or RNA-seq data analysis is easily obtained from Gene Expression Omnibus (GEO) [68–70], but the more highly expression genes are usually not consistent with the positive effects of enhancing cold-induced thermogenesis (or vice versa), possibly involving the negative feedback control or compensation effect. In this way, we identified a group of drugs in CMAP database that have higher scores of similarity (connectivity score) of gene-expression profiling as compared to the established enhancing activity in BAT and (or) sWAT signature, thereby potentially activating the UCP1 expression and inducing energy expenditure to combating obesity and associated diseases. Interestingly, rutin [50] and myricetin [52], both of which have been proved to have the capacity to elevate of UCP1 expression and enhance the thermogenesis in BAT and browning of sWAT, were also present in our results, indicating the feasibility of utilizing this system to screen drugs for stimulating UCP1 expression and elevating activity in BAT and (or) sWAT.

Further in vitro studies we focused on a previously unreported compound-indirubin, revealed that indirubin can enhance UCP1 expression as well as other thermogenic genes expression in differentiated brown adipocyte (Fig. 1e). These results demonstrated that indirubin could directly activate brown adipocytes in a cell-autonomous way. Two important characteristics of BAT or beige adipocytes are high mitochondrial DNA copy number and high rates of oxygen consumption. Our data showed that indirubin treatment significantly enhanced the mitochondrial OCR (Fig. 1f-e) in differentiated brown adipocytes, suggesting an elevated of the mitochondrial activities. Collected, our results suggest that indirubin can increase activity of the brown adipocytes by upregulating the BAT-enriched gene expression and by increasing mitochondrial activity.

We next focused on indirubin for further in vivo research. In the present study, our results showed that indirubin treatment remarkably reduced body weight gain and adiposity and improved whole-body metabolism in HFD mice, but there was no significant difference on normal chow diet conditions (Fig. 2). At the molecular level, we found that indirubin treatment in vivo resulted in higher UCP1expression levels (both in mRNA and protein levels) in BAT and sWAT (Fig. 6 and Fig. 7, respectively), which was in accord with the in vitro screening results (Fig. 1). Additionally, indirubin treatment also strongly upregulated the expression levels of BAT-enriched thermogenic genes involving in lipid metabolism and mitochondrial biogenesis, as well as the OXPHOS proteins in BAT and sWAT (Fig. 6 and Fig. 7). Importantly, indirubin treatment also caused browning effect on sWAT, as reflected by the increased expression of beige specific markers (*Tbx1, Cd137,* and *Tmem26*) in HFD-fed mice (Fig. 7e). These results suggest that indirubin normalized body weight and fat mass, which was likely involved in BAT activation and browning of sWAT, accompanied by increased energy expenditure and thermogenic gene expression in BAT and sWAT.

BAT also have a secretory role and BAT-derived endocrine factors (the so-called brown adipokines or batokines) have been thought to contribute to the systemic consequences of BAT activity. In particular, adiponectin plays a key role in mediating the benefits of BAT transplantation in rodents, including improvements in whole-body energy expenditure and glucose homeostasis [9–11]. Besides, previous research also reveals that patients with obesity and NAFLD exhibited a reduced adiponectin transcription in adipose tissue and decreased adiponectin concentration in plasma [71]. In addition, other moleculars, such as FGF21 and BMP, have also been suggested as exercise mimetics [72–74]. Interestingly, our present findings showed that indirubin treatment did not lead to obvious alteration in the expression levels of common adipogenic maker genes (*PPARγ2, Ap2*) in BAT and sWAT, but not adiponectin in BAT, which was increased significantly (Fig. 6d). In consist with this, the expression level of *Fgf21*was also significant up-regulated in differentiated brown adipocytes (Fig. 1e) and sWAT of mice under HFD after treatment with indirubin (Fig. 6d). Moreover, in our current work, we also found that indirubin improved systemic glucose and lipid homeostasis, ameliorated hepatic steatosis and obviously decreased the expression of inflammation-related genes in liver tissues induced by HFD (Fig. 5). The precise mechanism is not yet clear, even so, increased secretion of batokines after BAT activation and browning of sWAT, such as adiponectin and Fgf21, may also exert beneficial effects on treating type 2 diabetes and associated several metabolic comorbidities.

Finally, we further explored the underlying pathway by which indirubin upregulates UCP1 in adipocytes, which further enhances the activity of activity in BAT and (or) sWAT. It has been proved that increased PKA activity specifically in adipose tissue could upregulate UCP1 expression and ameliorate metabolism discords. PKA activates p38 MAPK and increases the expression of UCP1, thereby promoting thermogenesis in BAT [75, 76]. Moreover, increased PKA activity has also been linked to induction of browning in sWAT [77, 78]. In adipocytes, activated PKA increases the thermogenesis-related genes such as *Pgc1α* and *Ucp1* expression through phosphorylating the transcription factor CREB [22, 61]. PGC1α plays a key role in thermogenesis and oxidative metabolism. It has been reported that PGC1α promotes the expression of nuclear-encoded mitochondrial genes, thereby subsequently enhancing the mitochondrial biogenesis [61, 79]. Up-regulation of PGC1α expression in adipose tissues leads to a robust resistance to obesity and related diseases [80]. The activation of PKA triggers p38 MAPK phosphorylation, which also phosphorylates and activates of downstream effector PGC1*α* [75, 81, 82]. PGC1*α* then modulates the expression of UCP1, thereby promoting thermogenesis and browning in adipocytes [83–85]. Given that the PKA and p38 MAPK signaling pathway is critical for BAT activation and browning of (s) WAT, we next to evaluate whether indirubin treatment had any effect on PKA activity in adipocytes. Our study in vitro showed that indirubin significantly increased phosphorylation of PKA substrate (Fig. 8a). In particular, the present study showed that indirubin treatment could significantly increase the abundance of active phosphorylated forms of CREB and p38 MAPK in adipocytes. We also treated adipocytes with PKA inhibitor (H89) and p38 MAPK inhibitor (SB208503), respectively. PKA inhibitor or p38 MAPK inhibitor treatment clearly blocked the level of brown fat enriched mark proteins (UCP1 and PGC1*α*) (Fig. 8a-b). These observations could result from the increased activity of PKA and p38 MAPK in adipocytes, suggesting that indirubin activated BAT (as well as beige cell) via PKA and p38 MAPK dependent pathways.

It is interesting to notice that different compounds with even modest structure changes may lead to quite different physiological effects. One recent research has suggested that indirubin-3′-oxime (I3O), which is a synthesized analog of indirubin, has potential to prevent obesity and metabolic syndrome by inhibiting the differentiation of preadipocytes into mature adipocytes [86]. In our study, we noted that treatment of brown adipocytes with I3O in vitro can only displayed a slight but no significant increase in the expression of BAT marker gene *Ucp1* (Supplymentary Figure 2A), suggest different mechanism involved in the regulation of UCP1 expression compared with indirubin. Besides, our results showed that I3O markedly reduced the mRNA expression of genes related to activity and function of brown adipocytes (*Mcad, Lcad, Hsl, PGC1α*) in the differentiation of C3H10T1/2 cells, though I3O slightly but not significantly inhibited common adipogenic marker *Pparγ* (Supplymentary Figure 2B). Likewise, it should be considered that other closely related compounds, such as SB216763 and SB415286 (two GSK3 inhibitors), may also regulate one or both pathways controlled by indirubin in fat. Recent reports have indicated that small-molecule inhibitors of GSK3 have favorable metabolic effects in rodents, which exhibit some of the same metabolic effects as FGF21 administration, including improved glucose tolerance and prevention of diet-induced obesity [87–90]. One potential mechanism by which these agents could induce thermogenenic activation is by inhibitory phosphorylation of GSK3 in a PKA-dependent manner, which in turn result in enhanced activity of p38 MAPK signaling module [91]. In our study, we found treatment of differentiated brown adipocytes with indirubin (10 μM) indeed showed a modest inhibition of GSK3β by enhanced the phosphorylation of Ser-9 (p-GSK3β) (Ser9) in vitro (Supplymentary Figure 3). However, from our results, we are unable to conclude on the relative contribution of GSK3β or its paralogs to BAT activation. A precise mechanism by which indirubin treatment in adipocytes lead to increased thermogenesis remains to be further determined, but it is clear that PKA and p38 MAPK activation is very important for the effects. Nonetheless, the fact that indirubin treatment has a cell autonomous effect on BAT activation and browning of sWAT in vivo makes indirubin in particular a very promising drug candidate to protect against obesity and its related complications (Fig. 8d).

## Conclusions

This study we offer new clues for the indirubin, a purified compound obtained from the Indigo plant that decreased whole-body weight and adiposity, and improves glucose homeostasis and insulin sensitivity in HFD-fed mice. Specifically, screening potential UCP1 activators with the method of CMAP, which further enhance BAT activity and induce browning of WAT, could be a feasible therapeutic strategy for obesity and associated diseases.

## Supplementary information

**Additional file 1 Supplymentary Figure 1** (A-B) Cytotoxicity of indirubin upon induction in C3H10T1/2 cells before (48 h) (A) or after differentiation on day 6 (B).

**Additional file 2 Supplymentary Figure 2** RT-qPCR analysis mRNA expression of *Ucp1* and BAT-enriched genes in differentiated C3H10T1/2 cells on day 6. Data are presented as mean ± SD of six independent experiments performed in duplicate. ^*^p < 0.05, ^**^p < 0.01 compared with vehicle.

**Additional file 3 Supplymentary Figure 3** Western blot analysis performed with the indicated antibodies (p-GCK3β, GCK3β) in differentiated C3H10T1/2 cells on day 6.

**Additional file 4 Supplymentary Table 1** Primer Sequences used in this study.

**Additional file 5 Supplymentary Table 2** The enhancing CIT genes used as up-regulated genes and suppressive CIT genes from CITGeneDB.

## Data Availability

All data generated or analyzed during this study are included in this published article [and its Additional files].

## Abbreviations

CMAP: Connectivity Map
CITGeneDB: Gene data base of cold induced thermogenesis
CIT: Cold-induced thermogenesis
BAT: Brown adipose tissue
WAT: White adipose tissue
UCP1: Uncoupling protein 1
PGC: Peroxisome proliferator-activated receptor γ coactivator
VDAC1: Voltage-dependent anion channel 1
mtDNA: Mitochondrial DNA
NRF: Nuclear respiratory factor
Tfam: Mitochondrial transcription factor A
oxphos: Oxidative phosphorylation
RER: Respiratory exchange ratio
sWAT: Subcutaneous inguinal white adipose tissue
HFD: High-fat diet
NCD: Normal chow diet
GTT: Glucose tolerance test
ITT: Insulin tolerance tests
PKA: Protein kinase A
MAPK: Mitogen-activated protein kinase
FDG: Fluorodeoxyglucose
MGI: Mouse genome informatics
OCR: Oxygen consumption rate
PPAR: Peroxisome proliferator-activated receptor
CREB: CAMP response element-binding protein
GSK-3β: Glycogen-synthase kinase 3 β
