# Supplementary figures and images for: Indirubin, a small molecular deriving from connectivity map (CMAP) screening, ameliorates obesity-induced metabolic dysfunction by enhancing brown adipose thermogenesis and white adipose browning
Source: Nutr Metab (Lond). 2020 Mar 16;17:21. doi: 10.1186/s12986-020-00440-4 (PMC7076951; doi:10.1186/s12986-020-00440-4)

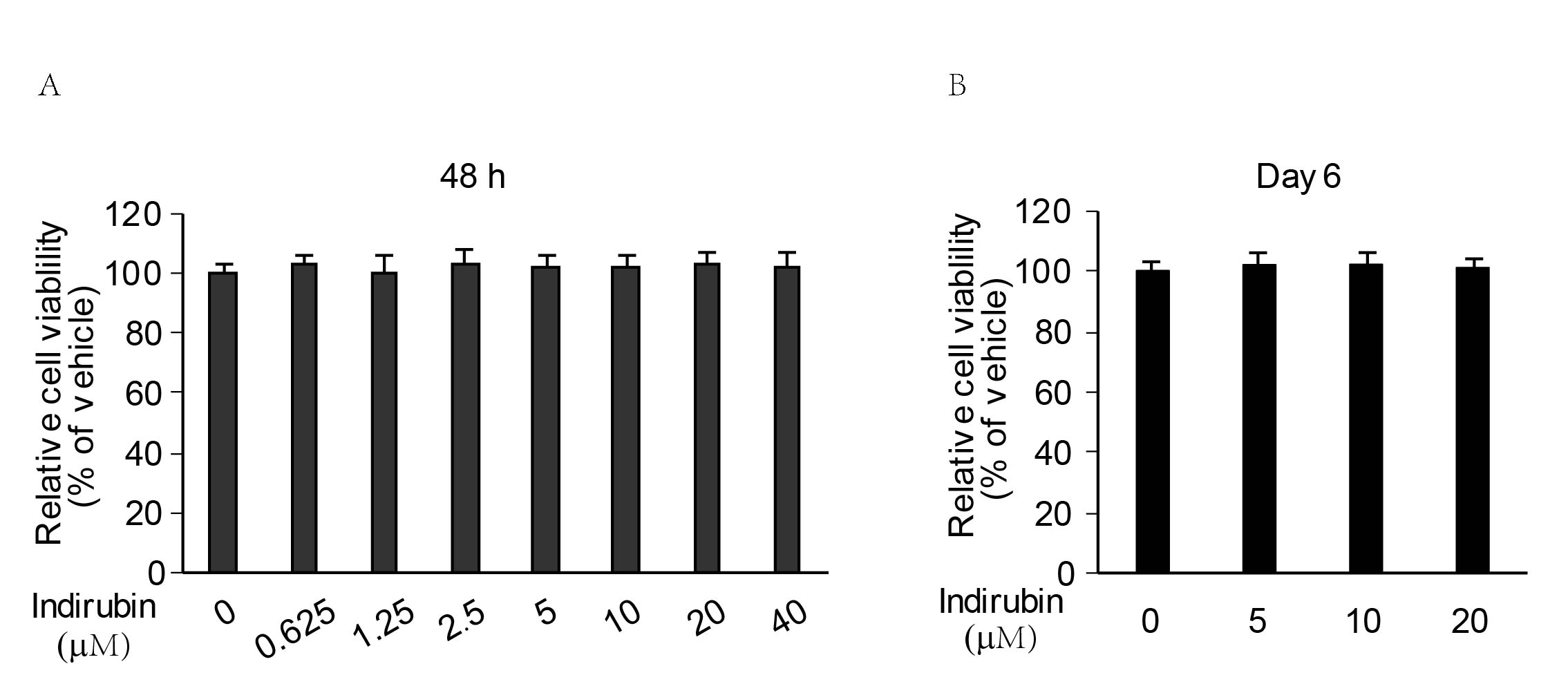

Supplement: Supplementary file 1 — Additional file 1 Supplymentary Figure 1 (A-B) Cytotoxicity of indirubin upon induction in C3H10T1/2 cells before (48 h) (A) or after differentiation on day 6 (B). [file 12986_2020_440_MOESM1_ESM.jpg]

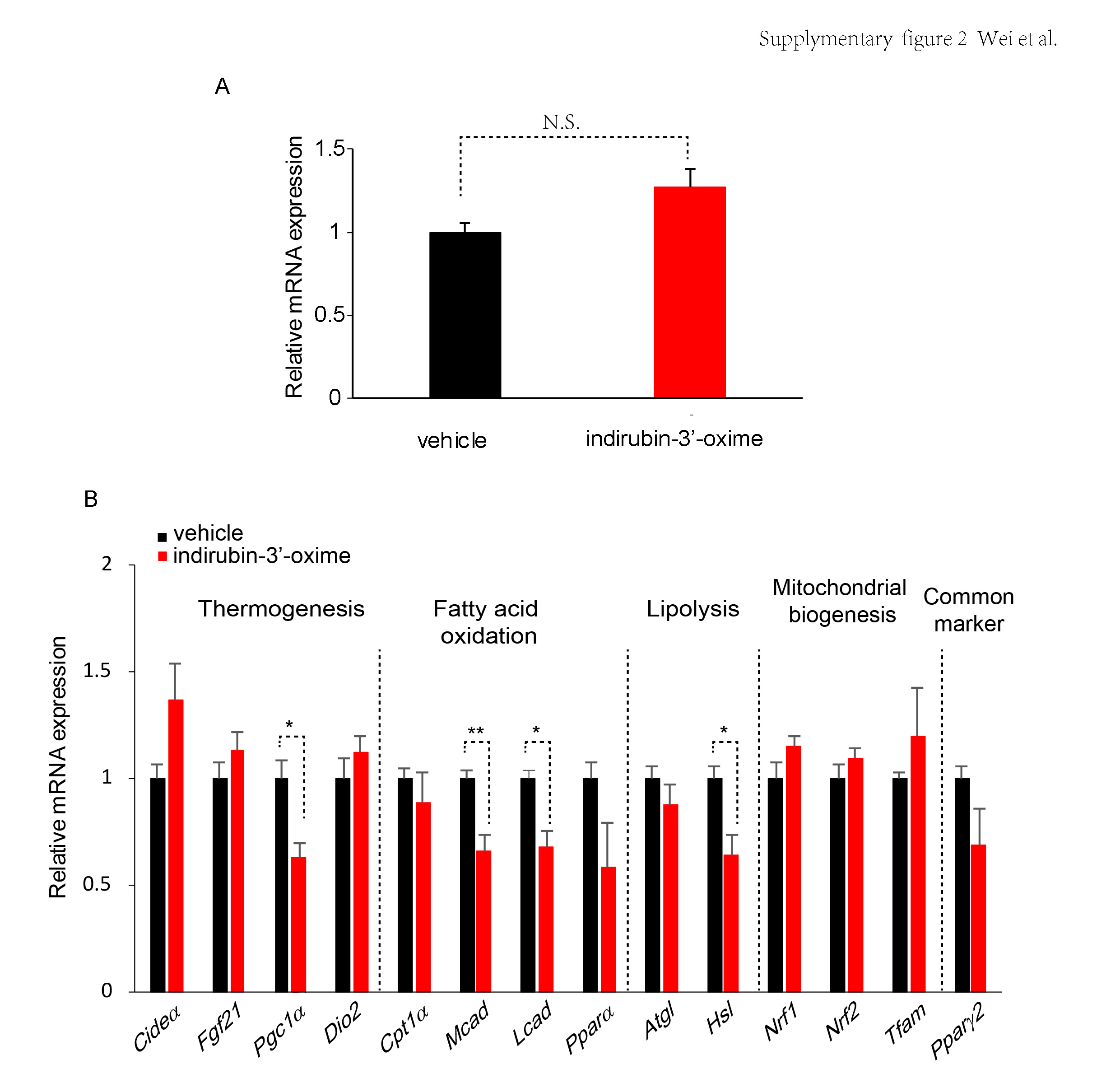

Supplement: Supplementary file 2 — Additional file 2 Supplymentary Figure 2 RT-qPCR analysis mRNA expression of Ucp1 and BAT-enriched genes in differentiated C3H10T1/2 cells on day 6. Data are presented as mean ± SD of six independent experiments performed in duplicate. *p < 0.05, **p < 0.01 compared with vehicle. [file 12986_2020_440_MOESM2_ESM.jpg]

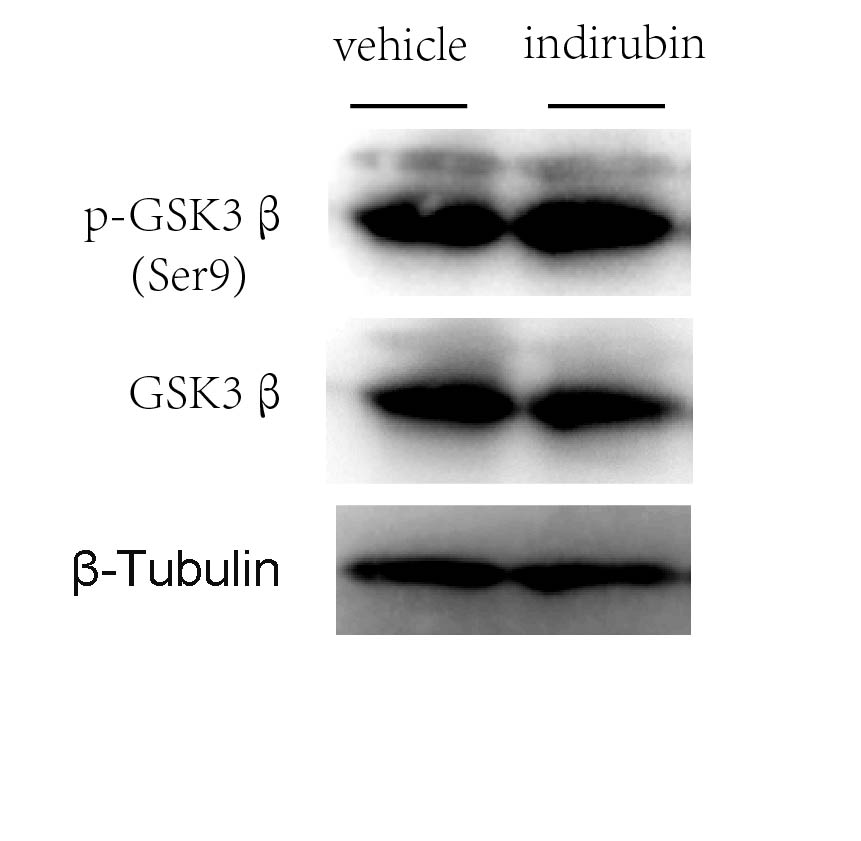

Supplement: Supplementary file 3 — Additional file 3 Supplymentary Figure 3 Western blot analysis performed with the indicated antibodies (p-GCK3β, GCK3β) in differentiated C3H10T1/2 cells on day 6. [file 12986_2020_440_MOESM3_ESM.jpg]
